# Osteoarthritis and microRNAs: Do They Provide Novel Insights into the Pathophysiology of This Degenerative Disorder?

**DOI:** 10.3390/life12111914

**Published:** 2022-11-17

**Authors:** Stefan Iulian Stanciugelu, Claudia Homorogan, Cosmin Selaru, Jenel Marian Patrascu, Jenel Marian Patrascu, Raymond Stoica, Diana Nitusca, Catalin Marian

**Affiliations:** 1 Doctoral School, Department of Biochemistry and Pharmacology, Victor Babes University of Medicine and Pharmacy, Pta Eftimie Murgu Nr. 2, 300041 Timisoara, Romania; 2 Orthopedic and Traumatology Clinic, Timisoara County Emergency Clinical Hospital, B-dul L Rebreanu Nr. 156, 300723 Timisoara, Romania; 3 Department of Orthopedics and Trauma, Victor Babes University of Medicine and Pharmacy, Pta Eftimie Murgu Nr. 2, 300041 Timisoara, Romania; 4 Department of Biochemistry and Pharmacology, Victor Babes University of Medicine and Pharmacy, Pta Eftimie Murgu Nr. 2, 300041 Timisoara, Romania; 5 Center for Complex Networks Science, Victor Babes University of Medicine and Pharmacy, Pta Eftimie Murgu Nr. 2, 300041 Timisoara, Romania

**Keywords:** OA, microRNAs, pathophysiology, diagnostic, biomarkers

## Abstract

Osteoarthritis (OA) is one of the most prevalent degenerative joint diseases in older adults and a leading cause of disability. Recent research studies have evidenced the importance of mi-croRNAs (miRs) in the pathogenesis of OA. In the present review, we focused on current literature findings on dysregulated miRs involved in the pathophysiology of OA. From the 35 case-control studies including OA patients compared to healthy controls, a total of 54 human miRs were identified to be dysregulated in OA. In total, 41 miRs were involved in the pathophysiological processes of OA, including apoptosis, inflammation, and proliferation, having either a protective or a progressive role in OA. The discovery of altered miR levels in OA patients compared to healthy controls determines a better understanding of the molecular mechanisms involved in the pathophysiology of OA and could open novel horizons in the field of orthopedics.

## 1. Introduction

Osteoarthritis (OA) is the most prevalent degenerative joint disease in older adults and a leading cause of disability [1]. Progressive breakdown of articular cartilage, remodeling of subchondral bone, chondrocyte hypertrophy, and synovium inflammation are clinically translated into pain, joint deformity, and malfunction of the affected joint [2]. Multiple etiological factors such as age, obesity, joint injury, mechanical stress, inflammation, and genetic factors are related to OA [3,4]. As several studies have already demonstrated, primary OA has a strong genetic component [5]. Recent evidence has made it noteworthy that epigenetic changes, altered expression of regulatory RNA, and its resulting gene expression modifications could also be involved in the biology of OA [6].

Epigenetics is the study of heritable phenotype changes that do not involve alterations in the DNA sequence [7]. Besides DNA methylation and histone modifications, microRNAs (miRs) are also linked with epigenetic mechanisms. Since the first human miR discovery, interest in this research field has grown tremendously, leading to an intense genomic investigation. MiRs are a class of small double-stranded non-coding RNAs, with a length of <25 nucleotides. They are transcribed in the nucleus to generate primary precursor molecules (pri-miRs), that undergo nuclear cleavage to form a precursor miR (pre-miR). The pre-miR is exported in the cytoplasm to create a miR duplex (miR:miR*) containing the mature miR. The miR base-pairs target messenger RNA (mRNA) to direct gene silencing via mRNA cleavage or translation repression based on the level of complementarity between the miR and the mRNA target [8]. Therefore, miRs regulate gene expression at the post-transcriptional level through translational inhibition or degradation of mRNAs [9]. 

In the past years, miRs were identified as being involved in various processes of cellular metabolism such as differentiation, proliferation, survival, apoptosis, embryogenesis, inflammation, and immune response [10]. They also appear to have a key role in tissue homeostasis [11] and in the pathogenesis of diseases and are dysregulated in several types of cancers [12], allergies [13], kidney disorders [14], psoriasis [15], psychiatric disorders [16], nervous system [17], and inflammatory diseases [18]. In OA in particular, the main function of miRs is to reduce the stability and translation level of their target mRNA involved in the pathophysiological processes, which includes inflammation, apoptosis, matrix synthesis, and chondrogenesis. MiRs seem also to modulate extracellular matrix deposition, and suppress or accelerate chondrocyte apoptosis [19,20]. A large number of studies have demonstrated the link between miRs and the pathogenesis of OA through the regulation of chondrogenic processes [21]. An imbalance between the catabolic and anabolic factors may be caused by abnormal expression of miRs, leading to cartilage degradation when cartilage homeostasis is disturbed [22].

Nonetheless, the exact biochemical mechanism of miRs in OA remains controversial, and information on the effects of these miRs are limited; therefore, in the present review, we focused on the current status of literature research on miRs involved in OA and reviewed the dysregulated miRs in OA, their function, and effective roles for a better understanding of OA pathogenesis.

## 2. Materials and Methods

### 2.1. Search Strategy and Study Selection

To find relevant articles dated through December 2021, a systematic search was performed in the PubMed, Web of Science, and Google Scholar databases using the following combination of keywords: “microRNAs” or “miRs”, “osteoarthritis”, “target gene”, “function”, “roles”, “pathophysiology” (all fields)” with the following enabled parameters: human, English language, articles published from 2010 to present. After searching the databases electronically, two independent reviewers performed an initial sort of the articles based on the titles. Duplicate references were excluded. In a second step, the abstracts and methodology of the studies were screened based on the inclusion and exclusion criteria.

### 2.2. Inclusion and Exclusion Criteria

The research articles inclusion criteria were: (1) case-control studies of subjects diagnosed with OA compared to healthy controls; (2) studies profiling miR expression levels of patients with OA and controls in human cartilage collected by surgery; (3) studies reporting cut-off criteria for determining whether miRs were differentially expressed between the OA and normal control; (4) original articles, published in the English language.

The research articles exclusion criteria were: (1) studies not conducted in human subjects (animal studies, in-vitro studies); (2) studies assessing other miRs, such as circ-miRs, lncRNA etc.; (3) non-original research papers, such as reviews, meta-analysis, pilot studies, abstracts, letters to editors; (4) duplicate studies; (5) studies written in other languages than English.

### 2.3. Data Extraction

**Figure 1:**
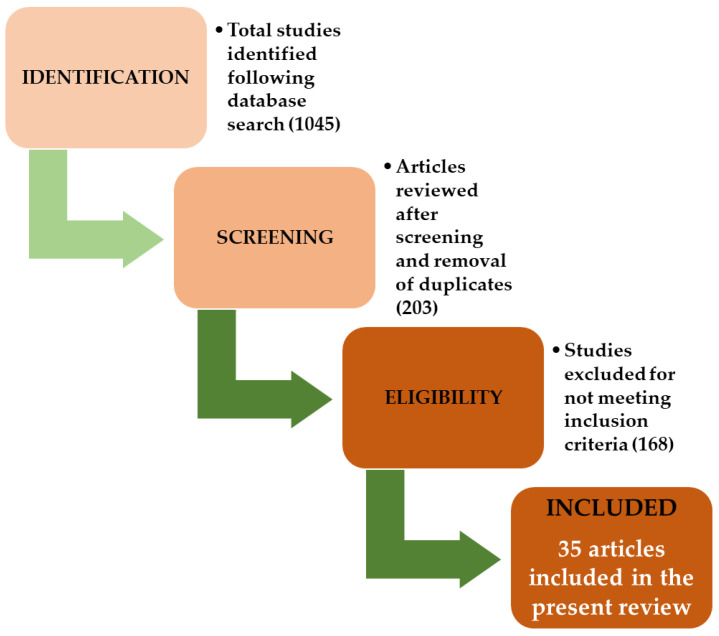
Flowchart of the study selection process.

At the initial search, 1045 articles were found. Considering the preliminary review of the titles and abstracts and excluding the irrelevant articles, a total of 203 studies were considered relevant. The validation process was determined by the authors after reviewing each full-text article separately. After full-text reading, 168 articles with irrelevant data were removed. In the end, a total of 35 studies published between 2010 and 2021 were selected to be included in the present literature review. Figure 1 presents a flowchart of the review selection process. After applying the eligibility criteria, two reviewers individually extracted the following information from the resulting studies: author, name of the study, the studied miRs and their direction of dysregulation (up- or downregulated), gene target and miRs function in OA.

## 3. Results

From the 35 included studies, a total of 54 human miRs were identified to be related to OA. 

### 3.1. MiRs Expression in OA Patients

Table 1 presents for each study the analyzed miRs and their dysregulation (up-regulated, down-regulated, or unchanged miRs) in patients suffering from OA compared to healthy controls. From the 54 differentially expressed miRs reported in the included studies, 25 were upregulated, 33 were downregulated, and 4 miRs had bidirectional dysregulation, both up-and downregulated (shown in bold).

### 3.2. MiRs Involvement in the Pathogenesis of OA

**Figure 2:**
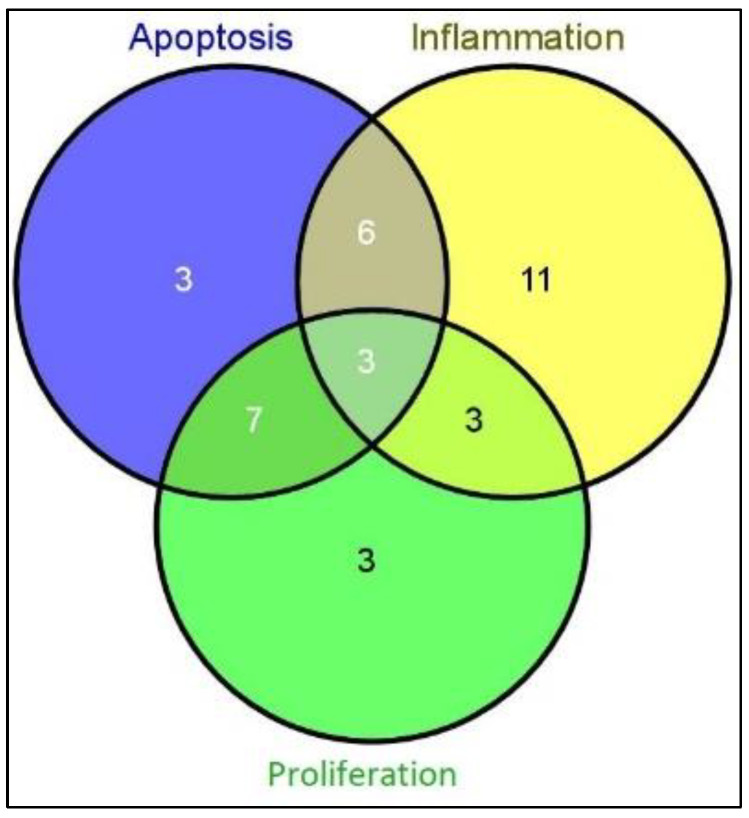
Venn diagram with the number of miRs involved in different pathophysiological processes.

MiRs appear to regulate the expression of target genes in OA cartilage, suggesting that they might be involved in the pathogenesis of OA through various mechanisms. It appears that apoptosis, inflammation, and proliferation are important processes involved in the pathophysiology of OA. The included studies provided hypothesized data on the pathophysiological effects of miRs in OA. Thus, 19 miRs are involved in apoptosis, 23 miRs are involved in inflammation, and 16 miRs play a role in cartilage proliferation (Table 2, Table 3 and Table 4 and Figure 2, respectively). 

From the 19 miRs involved in apoptosis (Table 1), 9 were also found to be involved in OA-related inflammation (miR-195-5p, -335-5p, -9, -145, -27b-3p, -125b, -140-5p, -455-3p, -142-5p, respectively), for which the information regarding the induced effect and signaling pathway can be found in Table 1. The remaining 14 miRs are only listed in Table 2, unfortunately with no available information regarding their pathophysiological effects (inflammation) in OA.

From the 16 miRs involved in cartilage proliferation, 10 were also found to be involved in apoptosis (Table 1, miR-195-5p, -335-5p, -337-3p, -26a-5p, -218-5p, -140-5p, -1236, -26b, -675-3p, -29b), with the remaining 6 miRs being involved only in proliferation. Unfortunately, information regarding their induced effect and signaling pathways is not yet available across references.

All miRs involved in all three processes (apoptosis, inflammation, and proliferation) can be seen in Figure 2.

From the total 19 miRs involved in apoptosis, only three of them were specific for apoptosis, 10 miRs were common with the proliferation process and 9 were also involved in inflammation. 16 miRs were involved in proliferation. Only 3 miRs were specific for proliferation and 6 miRs were common with inflammation. A total number of 23 miRs were related to inflammation, out of which only 11 were specific, and the other miRs were common for apoptosis and proliferation, as seen in Table 4.

### 3.3. Effective Roles of miRs in OA Pathophysiology

MiRs involved in OA pathophysiology (apoptosis, inflammation, and/or proliferation) seem to have either a protective or destructive role, the latter leading to the progression of the disease.

Table 5 shows the miRs and their positive or negative role in the pathophysiological process of OA. Inconsistencies across studies reveal that three miRs (miR-140-5p, miR-140-3p, and miR-146a, respectively) could have both protective and destructive roles in OA (shown in bold).

**Figure 3:**
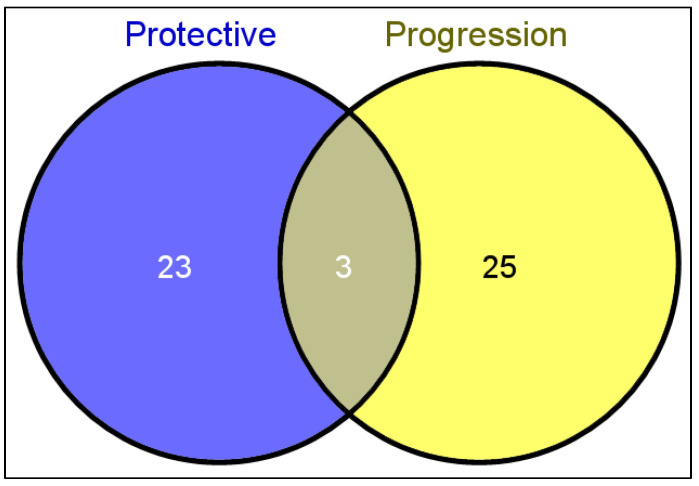
The number of miRs according to their effective role in OA.

Figure 3 shows that 23 miRs have only a protective role in OA and 25 miRs were involved only in the progression of OA. Three miRs can have both a protective and progressive role in OA: miR-140-3p, miR-140-5p, and miR-146a.

## 4. Discussion

In this study, we investigated the association between miRs and the pathophysiology of OA. Regarding miRs’ expression level, we found that miRs are generally more downregulated than upregulated in OA patients’ samples (articular cartilage, bone, synovial fluid and tissue, plasma, peripheral blood mononuclear cells (PBMCs), and whole blood) relative to healthy controls. MiRs seem to be involved in various pathophysiological processes of OA as well, such as apoptosis, inflammation, and proliferation; the great majority being involved in the inflammation process, and three miRs (miR-195-5p, miR-335-5p and miR-140-5p) being involved in all three processes. In addition, when studying their effector roles, miRs are displayed as having either protective roles, destructive roles, or (interestingly) both for miR-140-3p, miR-140-5p, and miR-146a.

It has previously been found that increased risk for OA development is associated with genetic and environmental factors, mechanical stress, fluid pressure, joint trauma, and inflammation. Several biological processes are involved in cartilage homeostasis, including proliferation, differentiation, inflammation, and apoptosis. Many of these processes appear to be regulated by different miRs. For example, miR-675-3p [23] and miR-203a [24] have been described as reducing inflammation and apoptosis. The proliferation of chondrocytes is also essential for maintaining the integrity of the articular cartilage [36]. In this context, recent studies have linked dysregulated expression of some miRs to several cellular processes, such as cartilage cell proliferation and differentiation. MiR-26a/b [37], miR-335-5p [25], miR-34a [38], miR-92a-3p [39], and miR-146a [26] seem to be involved in the pathogenesis of OA by regulating the expression of target genes related to chondrocyte proliferation. Namely, the downregulation of miR-197 in OA cartilage acts as a signal for chondrogenesis and promotes the proliferation and migration of cartilage cells by direct targeting of EIF4G2 [27]. Overexpression of miR-103 also promotes OA development by targeting and inhibiting the SOX6 gene. This miR has also been shown to inhibit chondrocyte proliferation and suppresses chondrocyte formation and maturation by reducing the expression of collagen II, glycosaminoglycan, and aggrecan [40]. In addition, up-regulation of miR-20 inhibits chondrocyte proliferation by directly targeting the ATG10 gene [28]. Duan et al. showed that miR-15a-5p promotes chondrocyte degradation by regulating the expression and function of PTHRP [29].

In addition, inflammatory cytokines such as IL-1-β, TNF-α, IL-6, and IL-8 are important mediators of the articular cartilage catabolic process. Several miRs such as miR-377-3p, miR-9, miR-98, mir-146, miR-27b-3p, and miR-675-3p appear to regulate IL-1-β, a key factor involved in chondrocyte apoptosis and matrix degradation, which is overexpressed in synovial fluid and OA cartilage. Interestingly, chondrocytes treated with this inflammatory cytokine have undergone matrix destruction and cartilage degradation [30,31]. Karlsen et al. have shown in an in vitro model of IL-1-β-induced OA that miR-140 inhibits the mediators of inflammation and cartilage degradation and also upregulates chondrogenic proteins [41]. Similarly, upregulation of miR-27a has a protective role against IL-1β-induced inflammation by reversing TLR4 gene expression [42]. miR-140 has been identified as protective against OA development by regulating cartilage development and homeostasis [32]. The regulatory role of miR-18a in chondrocyte differentiation has also been demonstrated [43].

Furthermore, chondrocyte apoptosis has also been shown to be part of OA pathogenesis, with apoptotic chondrocytes being more abundant in damaged cartilage than in smooth cartilage [44,45]. In this regard, upregulation of miR-1236 promotes OA progression through induced apoptosis and inhibition of chondrocyte proliferation [33]. Overexpression of miR-337-3p and of miR-455-3-p were shown to promote proliferation and inhibit apoptosis of chondrocytes in OA [46,47].

In addition to articular cartilage damage, OA is also associated with other pathological changes, such as synovial inflammation. Synovitis often occurs at different stages of OA and is associated with pain and joint dysfunction [48]. In synovial fibroblasts, upregulated miR-261-5p increased apoptosis, reduced inflammation, and decreased injury in vivo and in vitro [49]. MiR-16 and miR-132 were reported to be less expressed in synovial fluid of OA than in controls [50]. Let-7e, miR-454, and mir-885-5p [34] have even been identified as potential serum biomarkers for severe OA detection associated with synovitis. Synovitis-related, downregulation of miR-33b-3p, miR-140-3p, and miR-671-3p in serum OA has also been reported [35]. In contrast to previous studies, one study reported no differences in plasma miRs levels between OA patients and healthy controls [51]. The author reports some limitations to his study that may affect the final results, such as a prolonged freezing time period (up to 7 years) and the use of a lower amount of total RNA than is normally required by deep sequencing techniques.

Amongst the 35 studies included in the present review, only 4 of them were performed in biological tissues other than articular cartilage or bone of patients with OA. In general, the analyzed compartments were plasma, peripheral mononuclear blood cells (PBMCs) and synovial fluid. Interestingly, some miRs have been shown to have a heterogenous dysregulation among different tissues. For example, miR-146a was upregulated in PBMCs [52,53] and downregulated in chondrocytes [54]. By contrast, miRs derived from plasma and synovial fluid have been shown to have constant dysregulations: miR-155 [52], miR-34a-5p [38,55] and miR-16-5p [51] were upregulated in the same direction as the miRs derived from osteoarthritic cartilage, as shown in the study performed by Fan et al. for miR-155 [56], by Endisha H et al. for miR-34a-5p [38] and by Li L et al. for miR-16-5p [57].

The literature contains little information regarding the role of miRs as biomarkers for OA and their identification in comparison with other evaluation methods, such as radiographic imaging or MRI. The lack of evidence relies on the fact that miR levels are usually altered by coexisting comorbidities. In order to act as a biomarker for diagnosis, a miR should have high specificity and sensitivity and its analysis must be done as minimally invasive as possible. Among the advantages of miRs as biomarkers are high reproducibility (using RT-qPCR), their identification in body fluids, and their binding to proteins or exosomes, thus having a high degree of stability [58]. Some of the comorbidities display the same miRs as OA, thus reducing their sensitivity and specificity. Due to this fact, the role of miRs as biomarkers, especially in the early stages of both OA and early stages of other comorbidities is diminished since the miR concentration can lead to a false interpretation of the analysis, leading to a wrong conclusion and thus overlooking some of the pathologies and delaying their diagnosis and treatment. For example, high levels of miR-146a can also be identified in Type I and II Diabetes Mellitus and their associated complications and it is related to tumoral involvement in regulating metastatic turnover [59]. Furthermore, most of the studies are focused on miR concentration in synovial fluid once the OA process is ongoing, but their reliability as screening markers is diminished at the moment. Rousseau et al. conducted a study in 2020 and showed that miR-146 and miR-186-5p can be used as sensitive biomarkers in OA. Moreover, miR-146 levels were higher in grades 3 and 4 of OA, while miR-186-5p was a consistent biomarker in preclinical OA and its presence showed that the patients can develop clinical OA in the following four years [60].

As presented, miR related to OA can be found in blood, plasma, and synovial fluid; therefore, these fluids become the target for miR sampling. Many miRs can be found in various combinations between fluids, but there is no miR that can be found in every fluid. MiR-146-a was found in plasma and peripheral blood, miR-27 in synovial fluid; miR-27a-3p, miR-101-5p, and miR-378-5p were detected in synovial fluid and their detection was correlated with the late stages of OA [61]. Furthermore, Tavallaee et al. concluded that high concentrations of 23a-3p, miR-24-3p, miR-186-5p, miR-29c-3p, miR-34a-5p, and miR-27b-3p can be identified in synovial fluid in late-stage OA. Moreover, high levels of miR-155, miR-146A, miR-181a, and miR-223 were found in PBMCs when compared to healthy individuals [62,63]. Despite the fact that many miRs can be found in both synovium and serum, their display in serum is recognized as having inferior sensitivity. Xie et al. concluded that the serum-derived exosomes contain less miR information than synovial fluid exosomes; thus serum samples might be insufficient for an accurate identification and stratification risk. However, some clinicians might be reluctant at taking synovium samples, since it is a more invasive technique and it can lead to serious infectious complications and the risk might overcome the benefit; so the first choice for miR sampling might be serum taken from the peripheral blood [64].

In addition, miRs also represent a potential target for future therapies. At the moment their therapeutic role is very limited due to several factors: the first is finding the appropriate therapeutic agent to exert its effect upon a specific miR; second, an efficient delivery system that is minimally invasive and is highly efficient; currently the preferred delivery systems are parenteral or local route, miRs having a reduced absorption via oral administration. Lipid-based vesicles are used for delivery since they can easily cross the plasmatic membrane of the cells and have great tolerability and low cytotoxicity, but there are other available delivery systems such as nanoparticles, but with less efficiency and they present a certain degree of antigenicity; third, a therapeutic agent that has as few interactions as possible with other medications, has an acceptable level of toxicity, and has high bioavailability. Since one miR targets several genes, adverse effects must be taken into consideration when a specific miR is selected as a therapeutic target. In this case, local delivery (intraarticular) might be beneficial since the systemic effects should be reduced, and also a higher quantity of medication might reach the injury site without diluting it in the bloodstream. As presented, the levels and the type of miRs involved in OA differ as the pathology evolves. Specific therapeutic agents might not be as effective in the late stages as in the early stages because the levels of the targeted miRs change and the therapeutic effect might be diminished. Consequently, the therapy must be adapted accordingly to the present levels of miR, not only to have maximum efficiency, but also to avoid unnecessary side effects. The adjustment of the therapy requires continuous evaluation of miR levels [65].

Furthermore, other authors have reported similar reviews to our own, such as the study conducted by Zhang et al. (2017), where they mainly focused on the role of miRs in relationship with the pathogenesis of OA (via the regulation of articular chondrocytes’ gene expression), and on the potential of miRs as future therapeutic targets and diagnostic biomarkers for this degenerative disorder [66]. Mihanfar et al. (2020) conducted a review on the roles of exosomal miRs in OA, therefore focusing only on one type of sample specimen, possibly to reduce potential heterogeneity, and presenting only those miRs which were found to be encapsulated in microvesicles such as exosomes [67].

Nonetheless, there is one report assessing the roles of non-coding RNAs in different diseases with a focus on OA, but the authors merged together different molecules (such as miRs, long non-coding RNAs, circular RNAs, piwi-interacting RNAs, and small nucleolar RNAs) in a larger, more extended review, but less specific than our work [68]. On the other hand, Wang et al. (2022) reported a review on the role of long non-coding RNAs in OA, without mentioning any miR in their study [69]. Similarly, there is another report conducted by Kim et al. (2022) assessing the role of circular RNAs in knee OA, in an attempt to predict potential future therapeutic targets for OA [70].

Table 6 shows comparative data of common miRs found in both our research and different studies across the literature. Some of the miRs display different regulations, suggesting that there are many individual factors that contribute to their regulation.

Taken together, our review has several limitations worth acknowledging. Firstly, given the heterogeneity of the sample specimens that were used across studies, the direction of the expression level of the reported miRs was taken with respect to the highest trend. Secondly, due to a large degree of inconsistency across reports, we only listed the miRs with potential roles in the pathophysiology of OA, excluding from the table exact mechanisms in general, which were generally conflicting among reports. Thirdly, in vitro and animal model studies were excluded because of the biological differences between different animal species (mice, rats, rabbits, etc.) and human samples. Thus, differences in study designs, methodologies, and choice of statistics introduce a large degree of heterogeneity and bias across research studies, which makes the design of a review report a laborious task, and therefore the implementation of consensus regarding methodology guidelines and protocols is highly warranted.

## 5. Conclusions

In conclusion, understanding the exact roles and mechanisms of non-coding RNA species (such as miRs) in the pathogenesis of OA could bring new insights into disease management and future therapeutical approaches and improve the overall quality of life for patients suffering from this degenerative disorder.

## Figures and Tables

**Table 1:** Up- and down-regulated miRs in OA patients compared to healthy controls.

Upregulated miRs	Downregulated miRs
miR-195-5p	miR-335-5p
miR-9	miR-146
miR-25	miR-149
miR-98	miR-30
**miR-27a**	miR-337-3p
miR-34b	**miR-140**
miR-30b	**miR-146a**
**miR-140**	miR-27a-5p
miR-23a- 3p	miR-329
miR-24-3p	miR-655
miR-27a-3p	miR-708-3p
**miR-27b-3p**	miR-934
miR-29c-3p	miR-101-5p
miR-34a-5p	miR-20
miR-186-5p	**miR-27b-3p**
miR-483a-5p	miR-26a-5p
miR-103	**miR-27a**
**miR-146a**	miR-107
miR-218-5p	miR-222
miR-449a	miR-320c
miR-324-5p	miR-26a
miR-1236	miR-26b
miR-16-5p	miR-93-5p
miR-29b	miR-377-3p
miR-455-3p	miR-675-3p
	miR-16
	miR-132
	miR-33b-3p
	miR-140-3p
	miR-671-3p
	miR-140-5p
	miR-142-5p
	miR-197

**Table 2:** MiRs and their involvement in OA-related apoptosis.

Apoptosis	Induced Effect	Signaling Pathway	Reference No.
**miR-195-5p**	mRNA and protein levels of IL-1β, IL-6 and TNF-α were significantly enhanced	mir-195-5p activated the lPS-induced repression of the Wnt/β-catenin signaling pathway and activation of the nuclear factor (NF)-κB signaling pathway in ATDC5 cells	[5]
**miR-335-5p**	significantly reduces the expression of inflammatory factors (IL-1β, IL-6, and TNF-α)	significantly enhanced expression levels of the autophagy-related genes encoding the autophagy-related proteins Beclin-1, ATG5, and ATG7	[6]
**miR-9**	reduces the IL-1β mediated production of TNF-α and reduces basal and IL-1β-induced MMP13 protein release	PXR/RXR activation, G-protein coupled receptor (GPCR) signaling and Wnt/b-catenin signaling	[7]
**miR-145**	miR-145 up-regulation decreases LPS-induced inflammatory injury in ATDC5 cells through SAL	functioned in LPS-induced injury by blocking NF-κB and p38MAPK signaling pathways	[9]
**miR-337-3p**	-	miR-337-3p in OA increased PTEN expression and thus PI3K/AKT signaling pathway was blocked by PTEN	[13]
**miR-27b-3p**	TNF-αand IL-6 levels repressed by the silencing of PVT1 were significantly enhanced by knockdown of miR-27b-3p in C28/I2 cells treated by IL-1β	miR-27b-3p decreased cell viability that was promoted by the silencing of PVT1 in IL-1β-treated C28/I2 cells, miR-27b-3p abrogated the promoting effect of PVT1 knockdown on autophagy in C28/I2 cells challenged by IL-1β	[20]
**miR-125b**	-	miR-125b noticeably alleviated the LPS+pc-THRIL-induced JAK1/STAT3 and NF-κB pathways activation	[23]
**miR-26a-5p**	-	-	[24]
miR-107	-	miR-107 inhibited the activation of AKT/mTOR and NF-κB pathway	[25]
**miR-218-5p**	-	The expression of PIK3C2A, Akt, mTOR and S6 was downregulated, while 24 4EBP1 was upregulated	[26]
miR-222	-	-	[27]
**miR-140-5p**	miR-140-5p reduced the expression of HMGB1 protein, p-AKT (Ser473) and p-PI3K in IL-1β-induced chondrocytes	inhibited the PI3K/ AKT signaling pathway and suppressed the progression of OA through targeting HMGB1	[28]
**miR-1236**	-	miR-1236 overexpression promotes OA by inhibiting proliferation and induces apoptosis of chondrocytes, partially through PIK3R3 repression.	[29]
**miR-26b**	-	-	[30]
miR-93-5p	Overexpression of miR-93-5p was found to significantly inhibit IL-1β–induced chondrocyte apoptosis. In general, IL-1β enhances the expression of matrix-degrading enzymes (MMP3 and MMP13), which in turn degrade the ECM	miR-93-5p exerted its actions in chondrocytes partially through suppression of TCF4 expression	[31]
miR-675-3p	miR-675-3p overexpression inhibited the IL-6 and IL-8 expression that was caused by IL-1b stimulation	miR-675-3p mimic markedly attenuated the increase in the GNG5 expression levels	[32]
miR-29b	-	-	[33]
miR-455-3p	miR-455-3p significantly inhibited the viability of CHON-001 cells IL-1β-induced apoptosis of CHON-001 was significantly increased by up-regulation of miR-455-3p	miR-455-3p directly regulated COL2A1 expression through binding its 3′UTR sequence	[34]
miR-142-5p	miR-142-5p overexpression resulted in significantly decreased levels of IL-1β and TNF-α and increased levels of IL-10	miR-142-5p overexpression downregulated CXCR4 expression	[35]

The bold font style designates miRs with the most significant dysregulation between patients and controls across multiple studies (correlated).

**Table 3:** MiRs and their involvement in OA-related inflammation and proliferation.

Inflammation	Proliferation
miR-195-5p *	miR-195-5p *
miR-335-5p *	miR-335-5p *
miR-9 *	miR-140-5p *
miR-98	miR-337-3p *
mir-146	miR-26a-5p *
miR-145 *	miR-103
**miR-140-5p**	**miR-20**
miR-140-3p	miR-218-5p *
miR-146a	miR-320c
miR-155	miR-1236 *
miR-27b-3p *	**miR-26a**
miR-125b *	miR-26b *
miR-27a	**miR-377-3p**
miR-140-5p *	miR-675-3p *
**miR-20**	miR-29b *
**miR-377-3p**	**miR-197**
miR-455-3p *	
miR-142-5p *	
**miR-197**	
miR-23a-3p	
miR-24-3p	
miR-29c-3p	
miR-186-5p	

The bold font style designates miRs with the most significant dysregulation between patients and controls across multiple studies (correlated). The * symbol designates miRs which are also involved in apoptosis.

**Table 4:** The total number of miRs involved in pathophysiological processes of OA.

**Apoptosis (Total)**	**Inflammation (Total)**	**Proliferation (Total)**	**Total miRs in All Processes**
19	23	16	58
**Only apoptosis**	**Only inflammation**	**Only proliferation**	**Total miRs**
3	11	3	17

**Table 5:** MiRs’ effective roles in OA.

miRs with Protective Roles in OA	Effective Role	miRs with Destructive Roles in OA	Effective Role
miR-195-5p	inhibits chondrocyte apoptosis and the inflammatory response	miR-9	-
miR-335-5p	significantly increases cell viability and autophagy-related factors, and reduces inflammatory mediators	miR-145	miR-145 down-regulation increases LPS-induced inflammatory injury
miR-337-3p	inhibits the apoptosis of OA chondrocytes	miR-140	-
**miR-140-5p**	**inhibits inflammation mediators and cartilage degradation and upregulates chondrogenic proteins**	miR-30b	promotes cartilage degradation
**miR-146a**	**reduces the expression levels of inflammatory genes and catabolic proteases in nucleus pulposus**	miR-23a-3p	inflammatory stimulus
miR-27a-5p	-	miR-483-5p	severe cartilage damage, cartilage matrix degeneration, and increased chondrocyte hypertrophy
miR-20	inhibits chondrocyte proliferation and autophagy	miR-125b	promotes inflammatory injury
miR-103	-	miR-26a-5p	-
miR-222	induces osteoblast proliferation and attenuates cardiomyocyte hypertrophy	miR-27a	increases the proliferation, apoptosis and inflammation of chondrocytes
miR-26a	regulates chondrocytes apoptosis	miR-107	inhibits cell apoptosis and promotes autophagy in OA chondrocytes
miR-26b	regulates chondrocytes apoptosis	**miR-140-5p**	**increases cartilage degradation**
miR-93-5p	attenuates chondrocyte apoptosis and cartilage degradation	**miR-146a**	**increases and promotes inflammation**
miR-377-3p	alleviates chondrocyte apoptosis and cartilage degradation	miR-218-5p	promotes cartilage destruction
miR-16	-	miR-320c	promotes cartilage destruction
miR-132	-	miR-449a	promotes chondrocytes degradation
miR-33b-3p	-	miR-324-5p	-
**miR-140-3p**	**protective against OA development via inhibition of inflammation** **and oxidative stress, enhancement of autophagy**	miR-1236	induces cell apoptosis in chondrocytes, suppresses cell proliferation.
miR-671-3p	-	miR-16-5p	-
miR-675-3p	inhibits apoptosis, matrix degradation and inflammation of chondrocytes.	miR-29b	facilitates chondrocyte apoptosis
miR-142-5p	attenuates chondrocyte apoptosis and cartilage degradation	miR-34a-5p	promotes cartilage damage
miR-197	promotes chondrocyte proliferation, increases migration, and inhibits inflammation	miR-27b-3p	promotes cartilage damage
miR-329	-	miR-455-3p	enhances chondrocytes apoptosis and inflammation
miR-655	-	miR-98	promotes inflammation
miR-708-3p	-	**miR-140-3p**	**promotes inflammation**
miR-934	-	miR-24-3p	promotes inflammation
miR-146	negative regulator of immune responses	miR-29c-3p	promotes inflammation
		miR-186-5p	promotes inflammation
		miR-155	-

**Table 6:** Several miR expression levels in different studies compared to our own review.

MiRs	Regulation (Across Other Studies)	Regulation (Found in Our Review)	Reference
**miR-140**	↑	↑↓	[66]
**miR-146a**	↑	↑↓	[66]
miR-26a *	↓	↓	[66]
miR-26b *	↓	↓	[66]
**miR-27a**	↓	↑↓	[66]
**miR-27b**	↓	↑↓	[66]
**miR-9**	↓	↑	[66]
**mir-140**	↑	↑↓	[67]
miR-9 *	↑	↑	[67]
**miR-26a**	↑	↓	[67]
**miR-146a**	↑	↑↓	[67]
**miR-27a**	↓	↑↓	[67]

* designates those miRs which are consistent in our review compared to other review reports; “↑” symbol means upregulated and “↓” means downregulated (referring to miR gene expression).

## Data Availability

Not applicable.
